# Efficient expression of codon-adapted affinity tagged super folder green fluorescent protein for synchronous protein localization and affinity purification studies in *Tetrahymena thermophila*

**DOI:** 10.1186/s12896-015-0137-9

**Published:** 2015-03-25

**Authors:** Gürkan Yilmaz, Muhittin Arslanyolu

**Affiliations:** Graduate School of Science, Department of Advance Technologies, Biotechnology Program, Anadolu University, Yunusemre Campus, Eskisehir, Turkey; Department of Biology, Faculty of Science, Anadolu University, Yunusemre Campus, Eskisehir, Turkey

**Keywords:** Codon adaptation, Super folder GFP, *Tetrahymena thermophila*, Affinity purification, Protein localization, Dual tag

## Abstract

**Background:**

A superior Green Fluorescent Protein (GFP) mutant, known as superfolder GFP (sfGFP), is more soluble, faster folding, and is the brightest of the known GFP mutants. This study aimed to create a codon-adapted sfGFP tag (TtsfGFP) for simultaneous protein localization and affinity purification in *Tetrahymena thermophila*.

**Results:**

In vivo fluorescence spectroscopic analyses of clones carrying a codon-adapted and 6 × His tagged TtsfGFP cassette showed approximately 2–4-fold increased fluorescence emission compared with the control groups at 3 h. Fluorescence microscopy also revealed that TtsfGFP reached its emission maxima at 100 min, which was much earlier than controls expressing EGFP and sfGFP (240 min). A *T. thermophila* ATP-dependent DNA ligase domain containing hypothetical gene (H) was cloned into the 3' end of 6 × His-TtsfGFP to assess the affinity/localization dual tag feature. Fluorescence microscopy of the 6 × His-TtsfGFP-H clone confirmed its localization in the macro- and micronucleus of vegetative *T. thermophila*. Simultaneous affinity purification of TtsfGFP and TtsfGFP-H with Ni-NTA beads was feasible, as shown by Ni-NTA purified proteins analysis by SDS-PAGE and western blotting.

**Conclusions:**

We successfully codon adapted the N-terminal 6 × His-TtsfGFP tag and showed that it could be used for protein localization and affinity purification simultaneously in *T. thermophila*. We believe that this dual tag will advance *T. thermophila* studies by providing strong visual traceability of the target protein in vivo and in vitro during recombinant production of heterologous and homologous proteins.

**Electronic supplementary material:**

The online version of this article (doi:10.1186/s12896-015-0137-9) contains supplementary material, which is available to authorized users.

## Background

Homologous and heterologous expression of recombinant proteins in the unicellular ciliate *Tetrahymena thermophila* is frequently used in biological or biotechnological studies. The use of *T. thermophila* as an alternative eukaryotic host for the expression of recombinant proteins is based on some of its advantages, such as shorter cell division time, applicability of sterile cell culture techniques, maintenance of strains in liquid nitrogen, possibility of transformation using biolistic guns and electroporation, and the ability to introduce post-translational modifications, such as glycosylation [1,2]. However, *T. thermophila* has a disadvantage for heterologous protein expressions because of its use of an alternative codon dictionary with biased codon frequencies [3-5]. Additionally, the lengthy transformation protocol, which is associated with low transformation efficiency, needs to be improved to enable *T. thermophila* to become a more widely used eukaryotic host for recombinant protein production. The requirement for improved protein tags for protein localization in *T. thermophila* studies has triggered efforts to develop or adapt tags with different features. In *T. thermophila* studies, the most commonly used protein localization tag is the enhanced green fluorescent protein (EGFP), which is more stable and emits brighter fluorescence than wild-type GFP (WT-GFP). EGFP has been codon adapted for *T. thermophila* recently [6]. However, a novel, more advanced version of EGFP has been developed that needs to be adapted for *T. thermophila* studies.

The wild-type GFP (WT-GFP) is a ~26 kDa protein that emits green fluorescent light when exposed to the blue to ultraviolet spectral range [7,8]. The WT-GFP tag suffers from low fluorescence, comparative dimerization, aggregation, and high sensitivity to pH, making it inefficient. A variety of mutations have been introduced to make WT-GFP more soluble, stable, and have brighter emission characteristics [7,9]. GFP mutants have been widely used as visual marker proteins in developmental and cell biology studies [10,11]. Recently, a mutant, of sfGFP [GenBank accession no. HI069813.1 and Synthetic Sequence 3 from Patent EP1853717] was developed, which has superior features among GFP mutants, such as higher solubility, brighter fluorescence, faster folding, and higher resistance to denaturants such as urea and formamide [12]. These biochemical properties are attributed to the introduction of enhanced GFP mutations (F64L and S65T), cycle-3 GFP mutations (F99S, M153T, and V163A), and Super folder GFP mutations (S30R, Y39N, N105T, Y145F, I171V, and A206V) [12-14]. Hence, sfGFP also imparts solubility and enables proper folding of poorly folding fusion proteins [12,15]. Additionally, the introduction of a short affinity sequence, such as polyhistidine, to GFP resolves the lack of an affinity tag [16-20].

In some cases of heterologous protein expression, translation could be interrupted or terminated if the tag protein is used without codon adaptation. Therefore, tag and/or target gene sequences must be codon adapted to the host organism by the introduction of silent mutations [21-25]. The most commonly used protein localization tag in *T. thermophila* is a S65T mutant version of EGFP [3,6,10,11,26-32], which has recently been codon adapted for knockout studies in *T. thermophila* as a C-terminal protein localization tag [6].

To reduce the cost and experimental time of *T. thermophila* studies, this study aimed to develop an advanced dual fluorescence tag based on a *T. thermophila* codon-adapted sfGFP and an affinity tag such as 6 × His for simultaneous protein localization and affinity purification of the desired fusion protein. This type of dual tag will enable the tracking of protein production during in vivo and in vitro studies in *T. thermophila* studies.

## Results

### *Tetrahymena* codon adaptation of the Superfolder GFP gene

**Figure 1:**
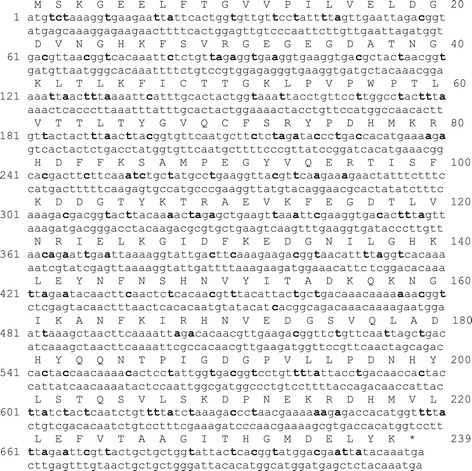
**Comparison of the DNA sequence of codon-optimized TtsfGFP with that of sfGFP.** Top DNA sequence is the codon-adapted TtsfGFP and the bottom sequence is that of sfGFP. The 126 mutated bases are shown in bold letters. There was no change in the amino acid sequence of sfGFP.

Analysis of the *Escherichia coli-*adapted synthetic sfGFP nucleotide sequence (encoding a 237 aa protein) showed that 57.72% of its codons (126 of 239 codons) or 21.2% of its sequence (153 of 714 bp) was not suitable for expression in *T. thermophila*. However, it was reported that the introduction of frequently used codons would help to improve gene expression [4,5]. Therefore, these non-adapted codons of sfGFP were re-assigned to *T. thermophila*’s frequently used codons as silent mutations, based on the codon frequencies, possible wobbling, and copy numbers of tRNA gene in the *T. thermophila* macronuclear genome, to avoid problems in protein expression [33]. The comparison of sfGFP and TtsfGFP sequences showed that more than 97% of the optimized codons (123 of 126 codons) had a change at the third position. However, only 20% of the changes (26 of 126 codons) were made at the first position, and changes in the middle base were limited to only two codons (Figure 1). The AT richness of TtsfGFP changed by only 6.1%, increasing from 57.8% to 63.9%.

### Codon-adapted TtsfGFP shows earlier and higher emission

**Figure 2:**
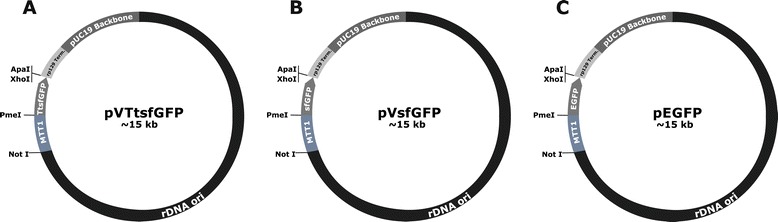
**Constructed protein expression vectors of** ***T. thermophila***. **A.** Vector pVTtsfGFP includes the *T. thermophila* codon-adapted TtsfGFP expression cassette; **B.** Vector pVsfGFP carries a non-codon-adapted sfGFP expression cassette; **C.** Vector pEGFP includes the non-codon-adapted EGFP expression cassette. All vectors were derived from pVGF. MTT1 is a cadmium-inducible promotor. The transcription is terminated using an rpL29 termination sequence.

**Figure 3:**
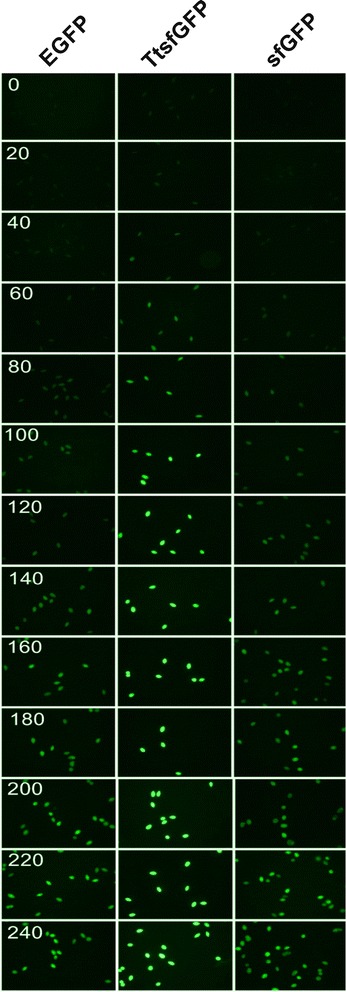
**Comparative in vivo fluorescence microscope analyses of EGFP, TtsfGFP, sfGFP expression.** The TtsfGFP-expressing cells emitted brighter green fluorescent light than the sfGFP and EGFP expressing clones at all period after induction with 2 μg/mL of CdCl_2_. A Leica DM6000 B fluorescence microscope equipped with a 20× objective and a GFP filter was used. Photo revision using PhotoScape 3.6.3 software was performed without any misleading changes because of the low quality of the images.

In this study, replacing the EGFP-Drp1 (Dynamin Related Protein 1) fusion gene in vector pVGF [10,34] with the 6 × His-TtsfGFP dual tag produced vector pVTtsfGFP (Figure 2). *T. thermophila* clones expressing TtsfGFP, sfGFP, and EGFP were grown and induced. Fluorescence microscopic analysis showed that TtsfGFP carrying clones began to emit fluorescence at 20 min, attaining maximum emission at 100 min (Figure 3). By contrast, sfGFP and EGFP carrying control clones began their emission at about 60 min, but did not reach the maximum level of emission comparable to TtsfGFP carrying clones until 240 min of incubation (Figure 3). The *T. thermophila* codon adaptation of sfGFP appeared to have increased the rate of protein synthesis rate and the overall expression level.

**Figure 4:**
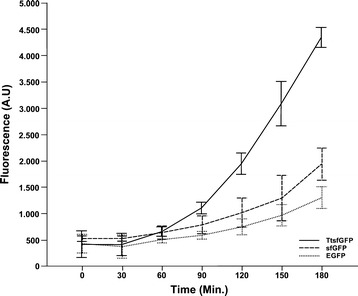
**Comparative in vivo fluorescent spectrophotometric analyses of cells expressing EGFP, TtsfGFP, and sfGFP.** Tetrahymena clones carrying TtsfGFP-, sfGFP-, and EGFP-expressing constructs were grown until the mid-logarithmic phase and cell density adjusted to 3 × 105 cells/mL. The cells were induced with 2 μg/mL of CdCl2. In vivo fluorescence spectrophotometric analyses were performed every 30 min at 488 nm excitation and 510 nm emission. The difference in emission between TtsfGFP, sfGFP, and EGFP began at 60 min and continued until 180 min. At the end of the time interval, TtsfGFP clones were found to emit ~2.2 fold and ~4 fold more fluorescence than sfGFP and EGFP, respectively.

Fluorescent emission spectral counting analyses showed that *T. thermophila* clones carrying TtsfGFP had ~2.2 fold and ~4 fold higher emission compared with clones expressing sfGFP and EGFP, respectively, at 180 min (Figure 4). However, the TtsfGFP-carrying clone showed much earlier acceleration of emission (beginning from 30 min) than the others. The distinguishable difference in fluorescence emission among the clones began after 60 min and remained until 180 min.

### Increased protein expression of *Tetrahymena thermophila* codon-adapted TtsfGFP tag

**Figure 5:**
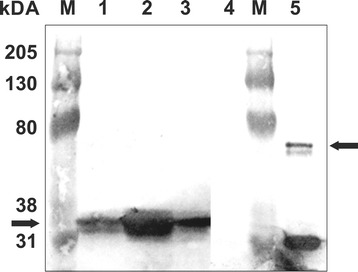
**Western blot analysis of total proteins from** ***T. thermophila*** **cells expressing TtsfGFP, sfGFP, or EGFP.** Equal amounts (30 μg) of EGFP (Lane 1), TtsfGFP (Lane 2), and sfGFP (Lane 3) were loaded. The left arrow shows a molecular mass of around 34 kDa corresponding to TtsfGFP. The quantity of TtsfGFP in the total protein extract appeared to be approximately 5–10-fold higher than sfGFP and EGFP. Total protein extracted from a *Tetrahymena* B2086 and CU428 cell mixture was used as a negative control (Lane 4). The positive control was TtsfGFP, which was constructed, expressed, and purified using Ni-NTA affinity purification from *E. coli*. The ~68 kDa band was predicted to be an sfGFP dimer (right arrow). Western blotting was performed with a monoclonal mouse anti-GFP antibody (1:1000). M: Bio-Rad Kaleidoscope western markers.

The total protein extracted from the induced *T. thermophila* clones at 180 min was analyzed by western blotting. Equal quantities of total protein (30 μg) from each clone were loaded onto SDS-PAGE. Western blotting was performed with a mouse monoclonal anti-GFP antibody as the primary antibody. TtsfGFP, sfGFP, and EGFP were observed as ~34 kDa proteins (theoretically molecular mass, 29 kDa, Figure 5, Additional file 1). The quantity of recombinant protein was the highest in the TtsfGFP clone compared with sfGFP and EGFP. These results demonstrated that using *T. thermophila* codon adaptation in the sfGFP gene improved translation efficiently.

### Macronuclear and micronuclear localization of a *Tetrahymena thermophila* hypothetical protein as a fusion protein with the TtsfGFP tag in *Tetrahymena thermophila*

**Figure 6:**
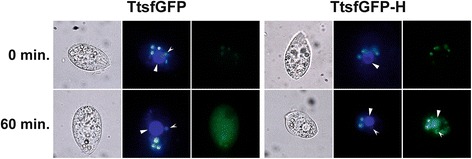
**The macronuclear and micronuclear localization of TtsfGFP-H fusion protein in** ***T. thermophila*** **.** pVTtsfGFP carrying *T. thermophila* cells as the control and *T. thermophila* carrying pVTtsfGFP-H cells were induced with 0.25 μg/ml of CdCl_2_ for one hour. 6 × His-TtsfGFP-H was localized to the macro- (arrowed large structure) and micronucleus (arrowed small structure). However, there was no detectable localization of the 6 × His-TtsfGFP tag protein except in the cytoplasm. The images were taken by a Leica DM6000 B fluorescence microscope equipped with a 63× objective. GFP filter was used for TtsfGFP (last column) and A filter for DAPI/Hoechst 33258 staining (middle column). The first picture columns were taken with light microscopy. The 2–3 vesicles that are seen in the cytoplasm are unknown structures.

A hypothetical gene containing a DNA ligase domain (H) was cloned into the C-terminal end of the 6 × His-TtsfGFP tag in pVTtsfGFP. After the simultaneous induction of *T. thermophila* cells carrying pVTtsfGFP-H and control cells carrying pVTtsfGFP with 0.25 μg/mL of CdCl_2_ for 1 h, microscopic analyses showed increasing GFP fluorescence in the Hoechst 33258 stained macronucleus and micronucleus of *T. thermophila* carrying pVTtsfGFP-H. However, in the control cells, a strong green fluorescence was emitted only from the cytoplasm, even after 1 h (Figure 6). Therefore, the nuclear localization signal sequence in the N-terminus of the hypothetical protein containing the DNA ligase domain (H), as predicted the CELLO program (Data not shown) [35], must be functional.

### Use of the 6 × His-TtsfGFP tag for affinity purification

**Figure 7:**
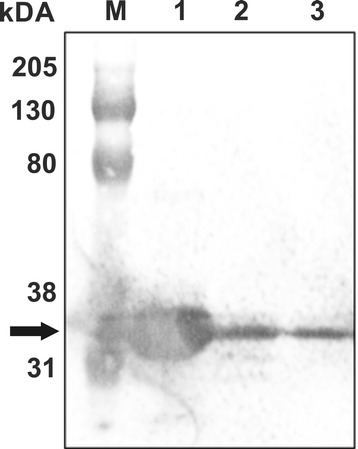
**Western blot analysis of 6 × His-TtsfGFP purified with by a Ni-NTA column.** The purified 6 × His-TtsfGFP isolated from cells with 2 μg/mL of CdCl_2_ for 18 h. From the Ni-NTA affinity purification columns, the recombinant 6 × His-TtsfGFP was observed as ~34 kDa protein in the first elution (Lane 1, arrow), the flow-through (Lane 2), and the first wash (Lane 3) determined using a monoclonal mouse anti-GFP antibody as the primary antibody(1:1000). M: Bio-Rad Kaleidoscope western markers.

**Figure 8:**
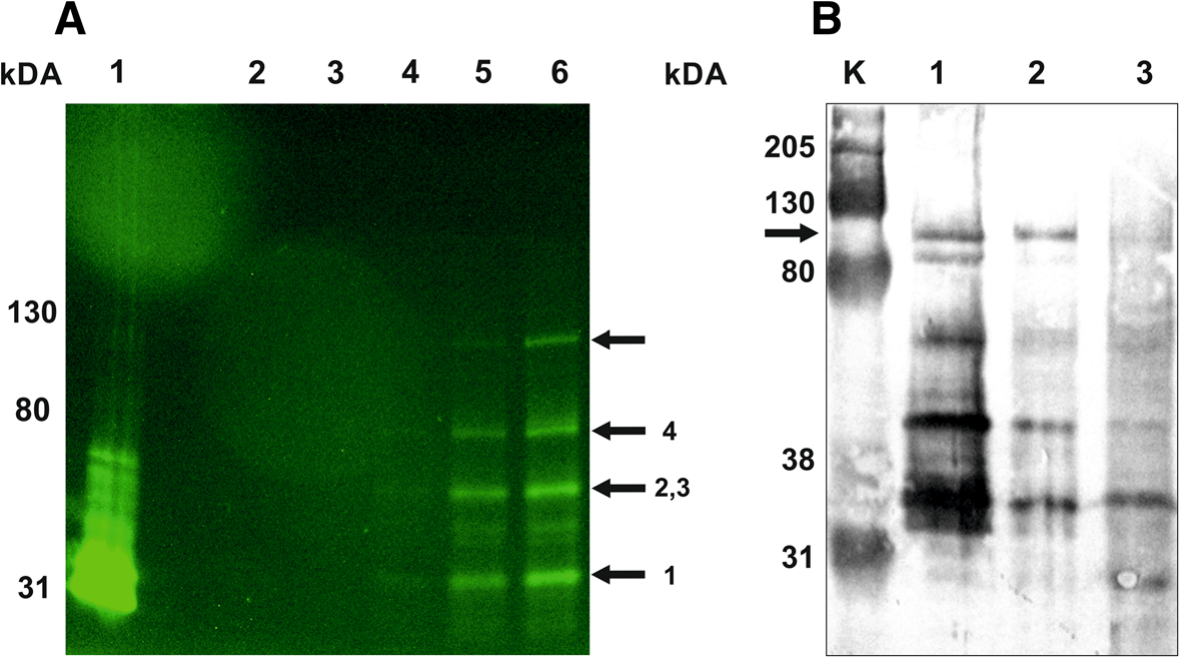
**Fluorescent and western blot analysis of the hypothetical ATP dependent DNA ligase domain containing protein. A.** SDS-PAGE was carried out in a discontinuous slab gel under semi-denaturing conditions by omitting mercaptoethanol from the sample buffer and without boiling. About 15 μg of total protein were loaded in each lane. Gels were visualized with Bio-Rad Gel Doc EZ using the blue sample tray for GFP. Lane 1: affinity purified 6 × His-TtsfGFP from *Eschericha coli*; lane 2: untransformed *T. thermophila* total cell protein (negative control); *T. thermophila* with pVTtsfGFP-H induced with 0.25 μg/mL of CdCl_2_ for 3 h; lane 3: zero time, lane 4: 1 h, lane 5: 2 h, lane 6: 3 h. **B.** The 6 × His-TtsfGFP-H fusion protein purified from *T. thermophila* pVTtsfGFP-H clone was induced for 18 h and analyzed with western blotting by using monoclonal mouse anti-GFP antibody (1:1000). The 6 × His-TtsfGFP-H was approximately 95 kDa (Lane 1, black arrow), as expected. Many fragmented proteins were also visible. Moreover, some of the target and fragmented fusion proteins were lost during washing (Lane 2) and flow-through (Lane 3) steps of Ni-NTA affinity purification. The predicted size of the fragments based on the rare codons plus 6 × His-TtsfGFP would be approximately 36.7 kDa (Arrow 1), 48 kDa (Arrow 2) and 50 kDa (Arrow 3). The roughly 70 kDa band could be dimer of these broken fusion proteins caused by the dimerization of sfGFP (Arrow 4). M: Bio-Rad Kaleidoscope western marker.

*T. thermophila* clones expressing the 6 × His-TtsfGFP tag were induced for 3 or 18 h with 2 μg/mL of CdCl_2_. The total soluble protein was extracted and the 6 × His-TtsfGFP was purified using an Ni-NTA affinity column. The ~34 kDa TtsfGFP protein was detected by anti-GFP antibodies upon western blotting of the affinity purified samples (Figure 7). These results suggested that the TtsfGFP could be successfully purified using Ni-NTA beads, although in a reduced amount (Figure 8).

In the *T. thermophila* cell line expressing the hypothetical gene encoding the ATP-dependent DNA ligase domain as a 6 × His-TtsfGFP-H fusion protein, the fusion protein was localized to the micro- and macronucleus (Figure 6). In western blotting using the Ni-NTA affinity purified lysate, the 6 × His-TtsfGFP-H fusion protein appeared as the predicted ~95 kDa protein, but with many short truncated fusion protein fragments, not only in the total protein (Figure 8-A), but also in the affinity purified samples (Figure 8-B). However, western blotting indicated that some target protein was lost during the wash and flow-through steps of affinity purification. These analyses nevertheless indicated that the 6 × His-TtsfGFP tag is suitable for the affinity purification of a fusion protein, acting as a tracking marker in column purification after the determination of its protein localization.

## Discussion

There have been attempts to develop better protein tags for recombinant protein production and functional studies in *T. thermophila*. For example, a *T. thermophila* codon-adapted version of EGFP was produced as a C-terminal epitope tag [28], but it was not evaluated for its translation and fluorescence efficiency [6]. Additionally, a potential dual affinity tag based on the *T. thermophila* glutathione S-transferase zeta and a 6 × His tag was reported [36]. In the present study, we developed an N-terminal affinity epitope tag using a more advanced GFP mutant, known as sfGFP, after codon adaption to *T. thermophila*. Cells carrying this new construct could be used not only for protein localization by fluorescent microscopy, but also for simultaneous affinity purification, and for protein function and protein-protein interaction studies. Indeed, we demonstrated that the *T. thermophila* codon preferences used in the 6 × His-TtsfGFP tag clearly improved its translational efficiency, resulting in an increase in the emitted fluorescence compared with the control groups (sfGFP and EGFP) (Figure 5). Additionally, TtsfGFP appears to possess superior properties compared with the controls, such as earlier and brighter fluorescence emission following induction with CdCl_2_. Other researchers have reported similar findings; i.e., that codon adaptation is necessary for efficient translation of heterologous proteins in *T. thermophila*. For example, the codon adaptation of human DNase-I and neomycin resistance gene (Neo4) improved their translation in *T. thermophila* [37,38]. These findings support the conclusion that codon adaptation increases the protein synthesis rate and the overall expression level of recombinant proteins in *T. thermophila* [23,24,39].

The utility of GFPs as protein localization tags may be severely compromised because of the absence of an affinity feature for protein purification, except for their immune precipitation with specific anti-GFP antibodies [28]. This deficiency can be overcome by the addition of a 6 × His-like affinity tags at appropriate positions in GFPs as N- or C-terminal poly-His affinity tag. Although this strategy has already been applied to GFPs used in *E. coli* and human studies [16-18,40], there has been no report on their use in *T. thermophila*. In this study, an N-terminal 6 × His tag, as a representative peptide affinity tag, was added to the codon-adapted TtsfGFP tag. This affinity feature of the 6 × His-TtsfGFP and 6 × His-TtsfGFP-H products was confirmed using Ni-NTA based affinity purification and western blotting. It is also possible that other peptide affinity tags, such as the Strep-tag II (WSHPQFEK) or the Calmodulin-tag (KRRWKKNFIAVSAANRFKKISSSGAL) could replace the 6 × His tag to further improve the affinity purification of TtsfGFP [12]. Moreover, microscopic analyses showed that the localization of 6 × His-TtsfGFP is clearly intracellular, whereas the 6 × His-TtsfGFP-H fusion protein was specifically localized in the macronucleus and micronucleus in *T. thermophila* (Figure 6), although there are number of short fusion protein products (Figure 8). From these data, one could easily propose a hypothesis that the hypothetical gene containing DNA ligase domain (H) must have a nuclear localization signal sequence in its N-terminal region, as predicted by the CELLO program [35] when analyzing the N-terminal 10, 24 and 143 amino acids or the full length amino acid sequence of H protein (Data not shown). These data together proved that localization and affinity purification of a target protein could be performed using the same protein expression construct, which would reduce costs and save time.

The fusion protein fragments in Figure 8 most likely resulted from incomplete translation and/or degradation of the protein. However, degradation of proteins is unlikely during short (1–3 h) and long (18 h) induction time because the size of the fragments were unchanged in both conditions (Figure 8). In addition, these fragments could not be produced by protein degradation because of the very short duration time on ice during the protein isolation. Moreover, Wu et al. reported that the presence of sfGFP helped the poorly folding TEV protease to fold properly and increased the yield of the TEV protease to ~22% [15]. Therefore, the presence of sfGFP in fusion proteins should reduce the amount of broken fragments because of the reduced folding stress. Conversely, the relatively low yield of Ni-NTA affinity purified 6 × His-TtsfGFP-H might be explained in two ways. First, the partially translated or degraded recombinant protein fragments could bind competitively to the Ni-NTA beads, thereby reducing the binding efficiency of the full-length 6 × His-TtsfGFP-H fusion proteins. Consequently, most of the fusion protein could have been lost during the washing steps as flow through (Figure 8-B; lane 2 and 3). Second, the absence of codon optimization of the homologous H gene may cause multiple pauses in translation, producing broken fusion proteins because of the presence of rare codons (lower than 2–3% in all genes), producing a 2.7 kDa peptide from CTG_24_ for Leu, a 14 kDa peptide from GCG_127_ for Ala and a 16 kDa peptide from CTG_144_ for Leu [22,23,33]. The size of the predicted broken fusion proteins based on the rare codons plus 6 × His-TtsfGFP could be about 36.7 kDa, 48 kDa and 50 kDa, which correspond to the sizes observed in Figure 8-A and -B. The approximately 70-kDa band could be dimer of these broken fusion proteins caused by dimerization of sfGFP (Figure 5 and Figure 8). Strong transcription of 6 × His-TtsfGFP-H fusion gene under the MTT1 promoter with an endogenously expressed H gene may lead rapid depletion of CTG and GCG tRNAs, causing ribosome stalling and finally inhibition of translation to produce these fragments [22,23]. Therefore, the codon adaptation of homologous genes, in our case the H gene, could be required to increase the recovery of the intact 95 kDa fusion protein for this type of fusion protein production. However, if the problem of contaminating protein fragments persists after codon optimization of the H-protein, the positioning of the affinity tag at the C-terminus of TtsfGFP-6 × His should be considered. The advantages such as a faster-folding, brighter emission and facilitator of the fusion protein folding and the codon adaptation of the sfGFP tag make TtsfGFP an alternative tag for protein localization and affinity purification in *T. thermophila* studies.

## Conclusions

In this study, we showed that the *T. thermophila* codon-adapted sfGFP mutant with an N-terminal 6 × His affinity tag was superior to EGFP and sfGFP, based on the improved translation efficiency, for simultaneous protein localization and affinity purification. Thus, we believe that this dual tag will help to advance *T. thermophila*-based studies by enabling target proteins to be visually traceable under in vivo and in vitro conditions. In addition, the 6 × His-TtsfGFP dual tag will enable proper folding and stability, and will extend the shelf life of target heterologous and homologous proteins expressed in *T. thermophila*.

## Methods

### Codon adaptation of Superfolder GFP and plasmid construction

Based on the codon use frequency, the absence and/or presence of tRNA gene(s), and gene copy numbers of *T. thermophila* [33], the sfGFP protein coding sequence was adapted by the introduction of silent mutations based on the *T. thermophila* codon dictionary (Figure 1).

Sequences encoding *T. thermophila* codon-adapted sfGFP (TtsfGFP) and non-codon-adapted control sfGFP were synthesized and cloned into a pUC57 vector with the addition of restriction and protease recognizing sites and 6 × Histidine affinity sequence by Shanghai Shine Gene Company (Molecular Bio-Technologies Inc., Shanghai, China).

The pVGF vector used in this study was a *T. thermophila* replicative protein expression vector carrying a paromomycin-resistant mutant rDNA origin and a Drp1 gene [10,34]. The gene was cloned under the control of the MTT1 promoter, which was inducible by CdCl_2_ [41].

The 6 × His-TtsfGFP and 6 × His-sfGFP coding regions were digested by PmeI-ApaI restriction enzymes from the pUC57 vectors and cloned into the pVGF vector after releasing the EGFP-Drp1 fusion gene. The vectors were named pVTtsfGFP and pVsfGFP, respectively. *T. thermophila* pEGFP, for use as a control, was derived from pVGF by deleting the Drp1 gene using XhoI and ApaI enzymes and re-ligating it with a linker carrying three stop codons (constructed by Küçükoğlu, N. and Arslanyolu, M.). Cell lines expressing sfGFP and EGFP were used as control groups.

A cDNA fragment of a *T. thermophila* hypothetical gene carrying an ATP-dependent DNA ligase domain (named “H” in this study, 1667 bp) [GenBank: XM_001011861.1] was used to test the localization feature of the TtsfGFP tag. cDNA fragments of “H” were digested with XhoI-ApaI restriction enzymes and cloned into the 3′ end of the 6 × His-TtsfGFP gene in the pVTtsfGFP, which was named pVTtsfGFP-H. NEB-10-Beta competent *E. coli* (C3019l, New England BioLabs Inc, Ipswich, MA, USA) were used in all cloning steps, and all vector constructs were confirmed by DNA sequencing (Quick Start Kit, 608120, Beckman Coulter CEQ8000,Brea, CA, USA). The Thermo GeneJet Plasmid MiniPrep kit (K0502,Thermo,Waltham, MA, USA ) and QIAquick Gel Extraction Kit (28706, Qiagene, Hilden, Germany) were used for plasmid DNA purification from *E. coli* and for extraction from agarose gels, respectively.

### Electroporation of *T. thermophila*

Electroporation of conjugant *T. thermophila* strains-CU428 and B2086, (Tetrahymena Stock Center, Cornell University, Cornell, NY, USA) was performed as reported previously, with minor modifications [42,43]. During conjugation, pairing efficiency was monitored until it reached at least 80% (usually ~2–3 h). Cells were pelleted and washed with 10 mM HEPES (pH 7.5) and re-suspended in 10 mM HEPES, such that there were 1.7 × 10^7^ cells/mL during the “macronuclear development stage 1” of conjugation (ca. 9–10 h after the start of conjugation). A mixture of 15–20 μg of plasmid DNA and 230 μL of cell suspension (in 20 mM HEPES pH 7.5) was placed in a Gene Pulser electroporation cuvette (0.4-cm gap), and pulsed with Bio-Rad Gene PulserXcell (440 V, 25-μF, 200 Ω; Hercules, CA, USA). Cells were incubated for 18–24 h for the recovery and execution of conjugation. After the addition of paromomycin at 100 μg/mL, the cells were incubated for 3 more days. This last step was repeated with gradually increasing concentrations of paromomycin (100–800 μg/mL) for 7–10 days. To assess the degree of transformation, 10 mL of cell suspension was induced with 2 μg/mL of CdCl_2_ [44] and observed under a fluorescence microscope to detect the emission of fluorescence (Leica DM6000, GFP HP Filter Cube 11532366; Wetzlar, Germany). The transformed cells were maintained in liquid nitrogen at −80°C until further use.

### Fluorescence microscopy and fluorescence analyses

To determine the comparative fluorescent emission times, TtsfGFP, sfGFP, and EGFP carrying transformed *T. thermophila* clones were grown until the mid-logarithmic phase in a PPY medium containing 100 μg/mL paromomycin at 30°C with 120 rpm agitation. After induction with 2 μg/mL of CdCl_2_, the clones were incubated in an orbital shaker at room temperature with 50-rpm agitation. Samples were drawn every 20 min, starting at time zero, and fixed with 0.5 μL of 20% formamide per 500-μL cell sample. The fixed clones were immediately photographed under a Leica DM6000 fluorescence microscope (GFP HP Filter Cube 11532366, 20× objective).

For protein localization studies, *T. thermophila* clones carrying the 6 × His-TtsfGFP-H fusion protein plasmid were grown in PPY medium (containing 100 μg/mL paromomycin) until the mid-logarithmic phase at 30°C with 120 rpm agitation. The cells were induced with 0.25 μg/ml of CdCl_2_ for low MTT1 promoter transcription [44]. The cells were fixed with 20% formamide for 1 h after induction and photographed using a Leica DM6000 fluorescence microscope (GFP HP Filter Cube 11532366, 40× objective). For fluorescence analyses, clones carrying plasmids expressing TtsfGFP, sfGFP, and EGFP were grown until the mid-logarithmic phase. The concentration of the cells was adjusted to 3 × 10^5^ cells/mL before induction. All the clones were induced with 2 μg/mL of CdCl_2_ and incubated at room temperature in an orbital shaker with 50-rpm agitation. After induction, 200 μL of samples were taken every 30 min, starting at time zero. Each sample was placed in a 96-well microtiter plate (Falcon 353915) and the fluorescence recorded at 488-nm excitation-510 nm emission in a Molecular Device Spektramax M2 [7,12]. The emission profiles of the *T. thermophila* clones were collected every 30 min for 180 min and the analysis was performed for three independent experiments.

## Additional file

Additional file 1:
**Expression of Recombinant TtsfGFP in** ***Escherichia coli.*** **In this study, codon adaptation of TtsfGFP was performed not only for** ***T. thermophila*** **, but also for** ***E. coli.*** Here, TtsfGFP and sfGFP (control) were cloned into the NdeI and BamHI sites of pET-16b. Recombinant 6 × His-TtsfGFP and 6 × His-sfGFP were expressed in *E. coli* BL21 (DE3). The **Figure S1.** shows Coomassie staining of the SDS-PAGE gel (Left gel): M. BioRad Standard protein marker, 1. Total protein of *E. coli* expressing 6 × His-TtsfGFP, 2. Affinity purified 6 × His-TtsfGFP, 3. Total protein of *E. coli* expressing 6 × His-sfGFP, 4. Affinity purified 6 × His-sfGFP. Ni-NTA affinity purification of 6 × His-TtsfGFP and 6 × His-sfGFP is shown in the right panel. 1E: first elution, 2E: second elution, and W: washing. These results showed that there is no difference in terms of recombinant protein production of TtsfGFP and sfGFP in *E. coli* BL21 (DE3).

## Abbreviations

PVR: Polymerase chain reaction
Bp: Base pairs
GFP: Green fluorescent protein
sfGFP: Superfolder GFP
TtsfGFP: *T. thermophila* sfGFP
EGFP: Enhanced GFP
H: *T. thermophila* ATP-dependent DNA ligase domain containing hypothetical gene
WT-GFP: Wild-type GFP
Neo4: Neomycin resistance gene 4
