# Supplementary material for: Efficient expression of codon-adapted affinity tagged super folder green fluorescent protein for synchronous protein localization and affinity purification studies in Tetrahymena thermophila
Source: BMC Biotechnol. 2015 Mar 25;15:22. doi: 10.1186/s12896-015-0137-9 (PMC4432788; doi:10.1186/s12896-015-0137-9)

### Additional file 1—Expression of Recombinant TtsfGFP in *Escherichia coli*

In this study, codon adaptation of TtsfGFP was performed not only for *T. thermophila*, but also for *E. coli*. Here, TtsfGFP and sfGFP (control) were cloned into the NdeI and BamHI sites of pET-16b. Recombinant 6×His-TtsfGFP and 6×His-sfGFP were expressed in *E. coli* BL21 (DE3). The figure (S1) shows Coomassie staining of the SDS-PAGE gel (Left gel): **M**. BioRad Standard protein marker, **1**. Total protein of *E. coli* expressing 6×His-TtsfGFP, **2**. Affinity purified 6×His-TtsfGFP, **3**. Total protein of *E. coli* expressing 6×His-sfGFP, **4**. Affinity purified 6×His-sfGFP. Ni-NTA affinity purification of 6×His-TtsfGFP and 6×His-sfGFP is shown in the right panel. **1E**: first elution, **2E**: second elution, and **W**: washing. These results showed that there is no difference in terms of recombinant protein production of TtsfGFP and sfGFP in *E. coli* BL21 (DE3).

Additional figure S1.


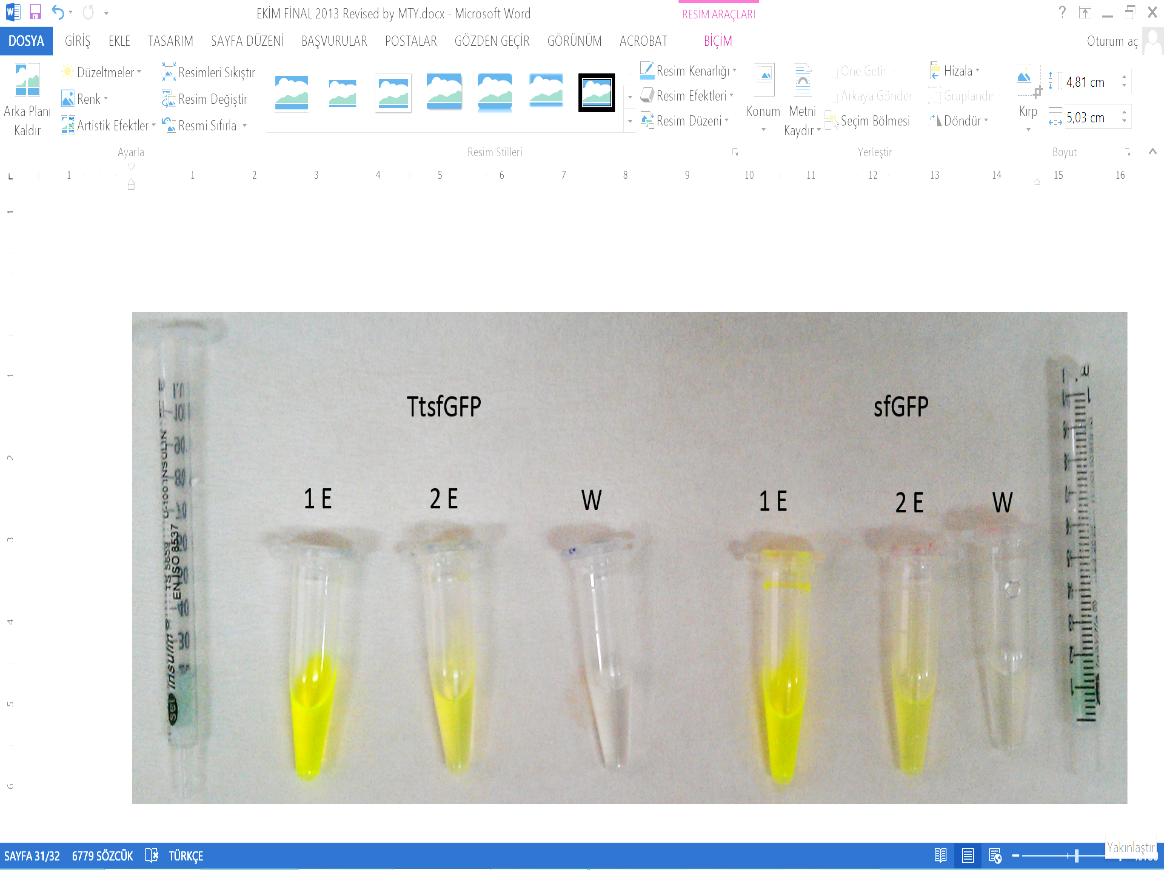

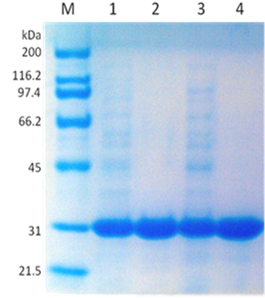

Supplement: Additional file 1: — Expression of Recombinant TtsfGFP in Escherichia coli. In this study, codon adaptation of TtsfGFP was performed not only for T. thermophila , but also for E. coli. Here, TtsfGFP and sfGFP (control) were cloned into the NdeI and BamHI sites of pET-16b. Recombinant 6 × His-TtsfGFP and 6 × His-sfGFP were expressed in E. coli BL21 (DE3). The Figure S1. shows Coomassie staining of the SDS-PAGE gel (Left gel): M. BioRad Standard protein marker, 1. Total protein of E. coli expressing 6 × His-TtsfGFP, 2. Affinity purified 6 × His-TtsfGFP, 3. Total protein of E. coli expressing 6 × His-sfGFP, 4. Affinity purified 6 × His-sfGFP. Ni-NTA affinity purification of 6 × His-TtsfGFP and 6 × His-sfGFP is shown in the right panel. 1E: first elution, 2E: second elution, and W: washing. These results showed that there is no difference in terms of recombinant protein production of TtsfGFP and sfGFP in E. coli BL21 (DE3). [file 12896_2015_137_MOESM1_ESM.docx]
